# NET Confusion

**DOI:** 10.3389/fimmu.2016.00259

**Published:** 2016-06-28

**Authors:** Natalia Malachowa, Scott D. Kobayashi, Mark T. Quinn, Frank R. DeLeo

**Affiliations:** ^1^Laboratory of Bacteriology, Rocky Mountain Laboratories, National Institute of Allergy and Infectious Diseases, National Institutes of Health, Hamilton, MT, USA; ^2^Department of Microbiology and Immunology, Montana State University, Bozeman, MT, USA

**Keywords:** host defense, extracellular trap, inflammation, inflammatory disorder, neutrophil

## Abstract

Neutrophils are arguably the most important white blood cell for defense against bacterial and fungal infections. These leukocytes are produced in high numbers on a daily basis in humans and are recruited rapidly to injured/infected tissues. Phagocytosis and subsequent intraphagosomal killing and digestion of microbes have historically been the accepted means by which neutrophils carry out their role in innate host defense. Indeed, neutrophils contain and produce numerous cytotoxic molecules, including antimicrobial peptides, proteases, and reactive oxygen species, that are highly effective at killing the vast majority of ingested microbes. On the other hand, it is these characteristics – high numbers and toxicity – that endow neutrophils with the potential to injure and destroy host tissues. This potential is borne out by many inflammatory processes and diseases. Therefore, it is not surprising that host mechanisms exist to control virtually all steps in the neutrophil activation process and to prevent unintended neutrophil activation and/or lysis during the resolution of inflammatory responses or during steady-state turnover. The notion that neutrophil extracellular traps (NETs) form by cytolysis as a *standard* host defense mechanism seems inconsistent with these aforementioned neutrophil “containment” processes. It is with this caveat in mind that we provide perspective on the role of NETs in human host defense and disease.

## Production of Neutrophils

Neutrophils are an essential component of the human innate immune response to bacterial and fungal infections. These leukocytes are among the first to be recruited to sites of inflammation and/or infection, and they are the most numerous white blood cell in humans. Under normal steady-state conditions, neutrophils develop from mitotic precursor cells (myeloblasts, promyelocytes, and myelocytes) in bone marrow for several days (~7.5 days) and then mature for 6–7 days as post-mitotic cells (metamyelocytes, band cells, and ultimately mature neutrophils) (1). Approximately 60% of the total nucleated cells in normal human bone marrow are granulocytes or granulocyte precursors (1). Cartwright et al. estimated the total granulocyte pool in bone marrow to be 1.86 × 10^10^ cells/kg body weight, of which 0.69 × 10^10^ cells/kg are mature neutrophils (2). A subsequent study by Dancey et al., which used a different method to label bone marrow granulocytes, reported the total number of bone marrow neutrophils as 0.77 × 10^10^ cells/kg body weight (3). With either method, it is clear that there is remarkable production of neutrophils in humans during steady-state conditions. Moreover, the production of granulocytes can be increased dramatically during severe infection – this process is known as emergency granulopoiesis (4).

The vast majority of granulocytes released from bone marrow into circulation are neutrophils (~95%), and these cells remain in circulation for a relatively short time (~12–18 h) (5). More recently, Pillay et al reported that the human neutrophil life span in circulation is 5.4 days (6), although other interpretations of these data have been proposed (7, 8). Consistent with high production of neutrophils in bone marrow, neutrophils comprise ~60% of leukocytes in human blood. Athens and colleagues reported that the total blood granulocyte pool comprises circulating and marginal granulocytes, which collectively are estimated as 6.5 × 10^8^ cells/kg body weight in total (3.2 × 10^8^ and 3.3 × 10^8^ cells/kg body weight for circulating and marginal granulocyte pools, respectively) (5). Several early landmark studies reported blood neutrophil turnover rate in humans as 0.87–1.63 × 10^9^ cells/kg/day (2, 3, 9). Thus, the estimated granulocyte turnover rate in humans is enormous – on the order of 0.5 – 1 × 10^11^ cells/day in a healthy adult (3, 5).

The continuous removal and replacement of neutrophils is critical for maintenance of immune system homeostasis and, importantly, the prevention of unintended damage to host tissues (10). Inasmuch as neutrophils contain and produce numerous cytotoxic molecules, which are highly effective at killing and degrading phagocytosed microbes, multiple (and redundant) mechanisms exist to prevent or limit host exposure to such molecules.

## Regulation of Neutrophil Turnover

Neutrophils traverse the vasculature in large numbers as an efficient means of readily disseminating to distal sites of host infection. Neutrophils are rapidly recruited to sites of infection by host and pathogen-derived molecules and have enormous pro-inflammatory capacity. The high rate of granulopoiesis frequently results in production of a superfluous number of neutrophils, and apoptosis is the predominant mechanism that regulates neutrophil turnover to maintain immune system homeostasis. In addition, neutrophils undergo apoptosis as a mechanism to limit pro-inflammatory capacity and to resolve infection. Neutrophil apoptosis is a highly regulated process mediated by several molecular mechanisms including intrinsic (intracellular) and extrinsic (extracellular) signaling pathways that lead to activation of caspase-3, and these pathways have been reviewed extensively (11–13). Constitutive (or spontaneous) neutrophil apoptosis is an example of the intrinsic pathway and governs removal of senescent cells, although the precise mechanism that triggers this process is incompletely defined. The intrinsic pathway is generally associated with cellular stress and mitochondrial damage and is mediated by members of the BCL-2 family of proteins. Extrinsic apoptosis is initiated by ligation of death receptors that bind FAS ligand, tumor necrosis factor (TNF)-α, or TNF-related apoptosis inducing ligand (TRAIL), and is largely at play within the context of complex inflammatory milieu.

Neutrophil apoptosis is a non-inflammatory process characterized by membrane blebbing, cell shrinkage, loss of cytoplasmic granules, cytoplasmic vacuolation, and nuclear chromatin condensation. Apoptosis is accompanied by DNA fragmentation into nucleosome-length fragments, exposure of phosphatidylserine on outer leaflets of the plasma membrane, and neutrophil outer cell membrane integrity is maintained throughout the process (14). Moreover, spontaneous neutrophil apoptosis is associated with diminished capacity for chemotaxis, degranulation, reactive oxygen species (ROS) production, and phagocytosis (14). Importantly, apoptotic neutrophils are safely removed by macrophages through a process known as efferocytosis (15–17). Macrophage recognition of apoptotic neutrophils is facilitated by receptors for phosphatidylserine, α_v_β_3_ integrin, and CD36 (18). Following recognition, macrophages phagocytose apoptotic neutrophils, and the process does not stimulate release of pro-inflammatory mediators (19).

Neutrophil lifespan is highly variable and can be influenced by many external factors capable of either prolonging survival or inducing apoptosis. A diversity of pro-inflammatory mediators, such as granulocyte-macrophage colony-stimulating factor (GM-CSF), interferon (IFN)-γ, interleukin 1β, C5a, and LPS, are known to delay neutrophil apoptosis (20, 21). Enhanced neutrophil survival presumably increases neutrophil numbers during early stages of inflammation and promotes clearance of bacterial pathogens from infected tissue. The process of phagocytosis significantly accelerates the rate of apoptosis in human neutrophils (22–24), and the increase occurs irrespective of any delay in cell fate imparted by cytokines or bacteria-derived factors (25). Effete neutrophils containing dead or partially digested microbes are cleared from infection sites by efferocytosis. Given that neutrophil apoptosis is accelerated by phagocytosis and apoptotic cells are at increased risk for necrotic lysis and/or leakage of cytotoxic molecules, efficient macrophage cell clearance is critical to prevent excessive damage to host tissue. Thus, the ability of pathogens to alter neutrophil fate by either promoting rapid lysis to eliminate neutrophils or interfering with efferocytosis is a plausible virulence strategy that can exacerbate acute inflammation. Indeed, bacterial pathogens such as *Streptococcus pyogenes* can additionally alter neutrophil apoptosis in a manner that ultimately results in rapid cell lysis (26) – a feature consistent with the ability of *S. pyogenes* to present clinically with necrotic lesions and gross inflammation (27). Moreover, it is known that some *Staphylococcus aureus* strains have the ability to promote rapid neutrophil lysis after phagocytosis (26, 28), and recent evidence indicates that the process occurs by programmed necrosis or necroptosis (29). Necroptosis is a pro-inflammatory form of cell death dependent on receptor interacting protein-1 kinase and leads to necrotic cell lysis. Thus, neutrophil apoptosis and efficient clearance by macrophages is essential for maintenance of host health, and pathogen-mediated deviations from this normal process that result in neutrophil lysis – irrespective of mechanism – contribute to pathogenesis.

## Control of Neutrophil Activation

The extraordinary ability of neutrophils to protect the host against a wide array of pathogens necessitates that these cells utilize highly toxic and damaging weapons to target pathogen incapacitation and/or destruction. Given the potential for collateral host tissue damage, it is essential that neutrophil activation is finely tuned to result in the appropriate level of response for any given situation. Indeed, neutrophils utilize a variety of mechanisms to control activation and subsequent delivery of these toxic components. One of the first approaches to controlling activation seems to be a very tight control of activation initiation. Resting neutrophils are maintained in the blood in an essentially dormant state, expressing very few, if any, adhesion molecules and receptors for activating ligands (30). However, these cells seem to be exquisitely sensitive to the presence of a danger signal or mechanical perturbation and can immediately increase their responsiveness through the mobilization of secretory vesicles, leading to surface expression of adhesion molecules, chemoattractant receptors, and other functional proteins involved in neutrophil mobilization without releasing potentially harmful inflammatory molecules (31). This reversible process is known as priming and transforms these cells into a state of heightened sensitivity and ability to generate a maximal host defense response (32). Indeed, the level of neutrophil priming has been linked to the severity of disease and disease outcome, and several studies have suggested that priming may be a good indicator of clinical disease activity (33, 34). On the other hand, absence of an infection or inflammatory stimulus would result in reversal of the primed condition back to a quiescent state, again demonstrating exquisite control of the neutrophil and its state of activation.

The selective mobilization of secretory granules during priming illustrates a second key mechanism utilized by neutrophils to regulate the inflammatory response. Neutrophils, also known as granulocytes, contain a number of cytoplasmic granules/vesicles that act as readily mobilizable reservoirs of potent enzymes and toxic molecules, which are selectively mobilized based on a hierarchy that is not completely understood but seems to control the level and types of enzymes released to meet the needs of the host defense situation (31). For example, gelatinase granules require a higher neutrophil activation threshold for exocytosis than do secretory vesicles, an even higher threshold is required for mobilization of specific granules, whereas the highest mobilization threshold seems to be for azurophil granules (31). Thus, selective compartmentalization of toxic and potentially host-damaging enzymes allows neutrophils to adjust their response to the level needed to address the insult by not inflict excessive damage to host tissues. Selective mobilization of granules also results in appropriate changes in the array of neutrophil cell-surface molecules and, thereby, modulates the way in which neutrophil interact with their environment.

As discussed above, neutrophil activation leads to the differential release of cytoplasmic granules, which participate in various host defense processes. Neutrophil activation is also characterized by the production of ROS *via* the activation of a multiprotein enzyme complex, known as the NADPH oxidase. This process, also known as the respiratory burst, results in the initial generation of superoxide anion $\left({\text{O}}_{2}^{\cdot -}\right)$; however, subsequent biochemical and enzymatic events can convert ${\text{O}}_{2}^{\cdot -}$ into more potent microbicidal products, including hydrogen peroxide (H_2_O_2_), a required substrate for the myeloperoxidase-halide system that generates hypochlorous acid (HOCl), hydroxyl radical (HO^⋅^), and other reactive oxygen and nitrogen species (35). While the NADPH oxidase system is essential for host defense, its products can also damage host tissues and, when inappropriately regulated, contribute to inflammatory disease (36). Thus, this system is also highly regulated through compartmentalization to avoid inappropriate activation and excessive host tissue damage. For example, the NADPH oxidase is composed of cytosolic and membrane-bound proteins that must assemble with each other through a complex sequence of signaling events, posttranslational modifications, and protein:protein binding interactions to finally achieve an active complex. Optimally, this complex assembles on the phagosomal membrane, where oxidants are targeted at high concentrations to a pathogen, but are also compartmentalized inside the cell to minimize host damage (37). Furthermore, neutrophil cytosol contains high levels of antioxidant enzymes, such as superoxide dismutase, catalase, and glutathione peroxidase to further limit release of toxic ROS into host tissues (38). Thus, it is clear that significant effort is devoted to the control of neutrophil activation and, thereby, unnecessary exposure of the host to damaging agents through regulated priming and activation, sequential mobilization of cytoplasmic granules, and compartmentalization of effector systems.

## Neutrophils and Inflammation

Inflammation is a host protective response against invading microbes or tissue injury. It consists of complex interactions between soluble mediators and host cells with hallmark features that include swelling, redness, pain, and heat. During acute inflammation, initial recognition of pathogen or damage-associated molecular patterns by pattern recognition receptors on tissue resident immune cells elicits production of immune mediators (39). Subsequently, these immune mediators (e.g., pro-inflammatory cytokines, chemokines, eicosanoids, and vasoactive amines) create a chemoattractant gradient that primes neutrophils and summons these cells to the site of injury or infection. This process is accompanied by vascular permeability and increased expression of selectins on activated endothelium, which, in turn, increases neutrophil adhesion and extravasation (40). Upon arrival in the infected tissues, neutrophils phagocytose and kill microbes using processes described above. Additionally, neutrophils secrete numerous pro-inflammatory molecules that amplify the immune response, and exocytosed granule proteases contribute to extracellular matrix degradation and tissue remodeling (41–43).

Non-phlogistic removal of effete and/or apoptotic neutrophils by mononuclear phagocytes is crucial to the resolution of inflammation and initiation of the tissue repair process (44–46). The overall importance of macrophages in tissue repair and restoration of homeostasis is perhaps exemplified by a mouse wound-healing model, in which depletion of these cells results in impaired angiogenesis, reduced granulation tissue formation and collagen deposition, decreased cell proliferation, and delayed re-epithelialization (47). Thus, under normal circumstances, acute inflammation is a self-limiting process that eliminates invading microbes and promotes tissue repair and return to homeostasis. Eicosanoids and other lipid molecules play a key role in the initiation and resolution of the inflammatory response (48). For example, leukotrienes and prostaglandins such as PGE_2_ are essential for trafficking of neutrophils to sites of infection. On the other hand, high concentrations of PGE_2_ in inflammation exudate signals for host activation of the 15-lipooxygenase pathway and lipoxin production, which stop recruitment of neutrophils and promote the resolution of inflammation (49, 50). Lipoxins belong to a group of specialized pro-resolving mediators that includes resolvins, protectins, and maresins. These lipid mediators promote recruitment of monocytes, efferocytosis of apoptotic neutrophils, uptake of debris, resolution of inflammation, and tissue regeneration (51, 52). Interestingly, generation of resolution signals starts early during the inflammation process and often depends on cell–cell (e.g., neutrophil–endothelial cell) interaction (50, 53).

It is widely known that neutrophils play a key role in inflammatory diseases. When the intricate network of signals controlling inflammation becomes imbalanced or the acute inflammatory response fails to eliminate the source of tissue damage, it can transform into a chronic inflammatory state. During chronic inflammation, the majority of tissue damage is caused by macrophages, monocytes, and granulocytes (54–57). Rheumatoid arthritis (RA) is an example of a chronic inflammatory disease to which the contribution of neutrophils has been studied extensively. Interestingly, neutrophils isolated from patients with RA are primed for ROS production and resemble low-density granulocytes (LDGs) from lupus erythematosus patients (58, 59). Production of ROS and release of granule enzymes by neutrophils contribute directly to cartilage and joint damage and perpetuate the inflammatory response (60).

Host tissue damage can also be caused by neutrophils during the acute inflammatory response. For example, neutrophils are known to contribute directly to lung tissue damage during severe pneumonia caused by *Staphylococcus aureus* (61, 62). This severe tissue damage, which in humans can be fatal, is largely caused by cytotoxic molecules released from activated and lysed neutrophils (63, 64). Inasmuch as neutrophil-derived cytotoxins are central to the pathology of inflammatory diseases, it should not be surprising that neutrophil extracellular traps (NETs), which are largely reported to form from a cytolytic process, are associated with many diseases or pathologic conditions.

## NETs and Disease

**Figure 1:**
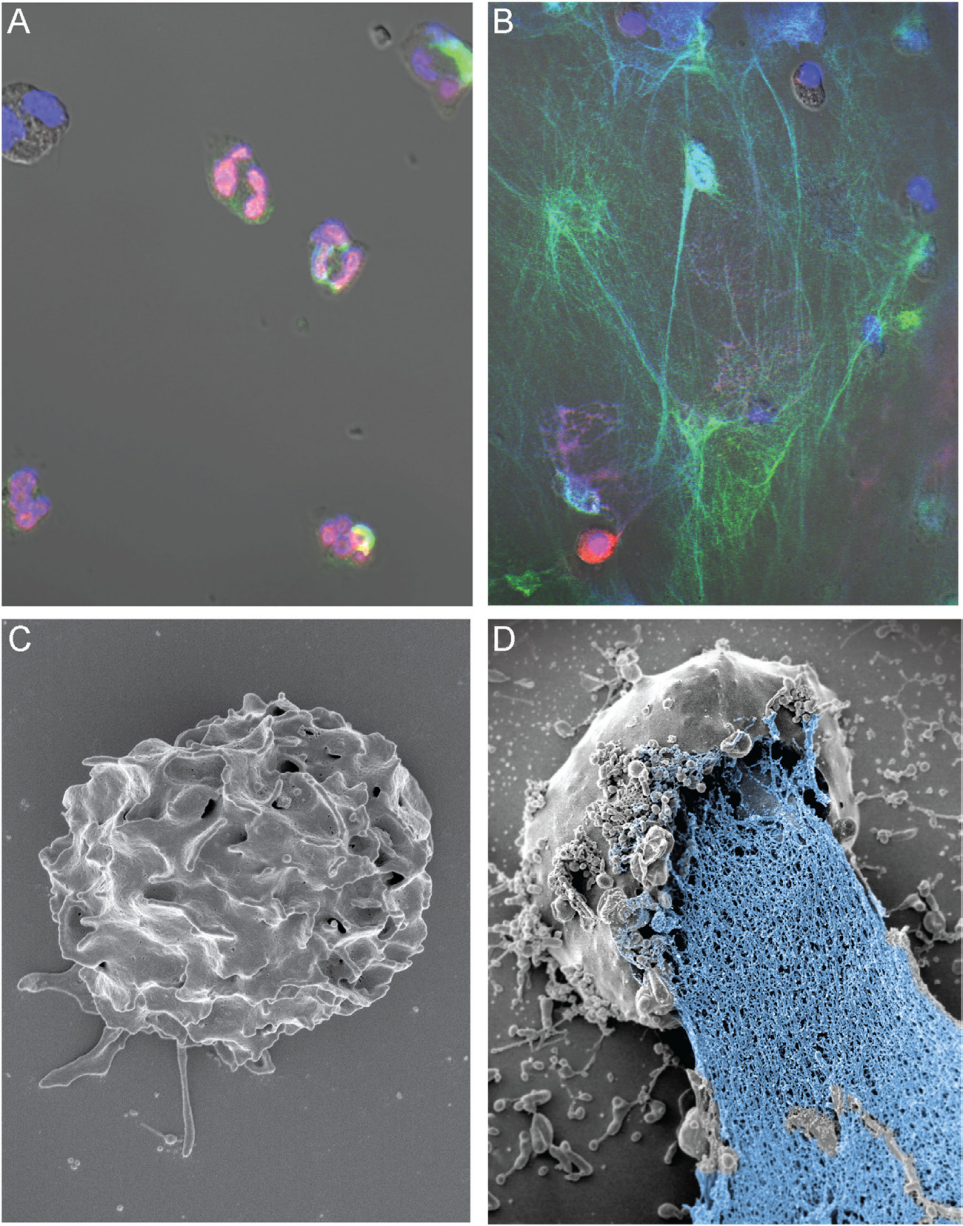
**NETs form during osmotic lysis of human neutrophils**. **(A)** Immunofluorescence staining of freshly isolated human PMNs (histone 2A; red), MPO (green), and DNA (DAPI; blue). **(B)** NETs formed following electropermeabilization (pulse of 800 V at 25 mF). Brightness and contrast of the images in **(A,B)** were adjusted in Adobe Photoshop CC2014 (Adobe Systems Inc., San Jose, CA, USA). **(C)** Scanning electron micrograph of a control neutrophil that was not electropermeabilized, and **(D)** NET-forming human neutrophil following electropermeabilization (pulse of 600 V at 10 mF). Studies with human neutrophils were performed according to a protocol approved by the Institutional Review Board for Human Subjects, US NIAID/NIH, as described elsewhere (87). All subjects gave written informed consent prior to participation in the study and in accordance with the Declaration of Helsinki. The image in **(A)** was originally published in Ref. (87). Copyright © (2013) The American Association of Immunologists, Inc.

Neutrophil extracellular traps are filamentous web-like structures that consist of extruded nuclear DNA and histones and are decorated with neutrophil granule enzymes, such as MPO, elastase, cathepsin G, and lactoferrin (65). They can be formed in response to infectious agents, inflammatory mediators, and/or under certain conditions, including non-specific osmotic cytolysis (Figure 1). NETs have been reported to entrap and kill numerous microorganisms (66–71). Many studies have investigated the molecular events leading to the formation of NETs. The first cell death mechanism proposed to explain formation of NETs was named NETosis (72), and the authors reported that it is RAF/MEK/ERK pathway dependent and requires ROS production (72). During NETosis, ROS trigger release of elastase from azurophilic granules into the cytoplasm, which then translocates to the nucleus and promotes decondensation of the chromatin through degradation of histones (72–76). This process is followed by rupture of the plasma membrane and extrusion of the DNA granule–protein complexes into the extracellular milieu to form NETs. Recent studies have compared signal transduction events involved in necroptosis and PMA-induced NETosis, but the findings of two studies were discordant (77–79). Not all reported mechanisms of NET formation require NADPH oxidase or cell lysis. One of the NADPH oxidase-independent mechanisms for NET formation was reported to be a calcium-ionophore-mediated process that utilizes mitochondrial ROS (80). Yousefi et al. reported that NETs form by release of mitochondrial DNA and that this process is not associated with cell death or lysis (81). A similar phenomenon has been described for eosinophils (82, 83). Kubes and colleagues made the intriguing discovery that neutrophils form ETs by extrusion of nuclear DNA, while the cells remain intact and functional afterward (84, 85). This process has been called vital NETosis – although the term “vital NET release” is perhaps less confusing (86). Such a process would circumvent many of the potential issues associated with cytolytic NET formation. However, the vast majority of studies report NETs formed by cytolysis.

Although the process of NET formation *in vitro* is relatively well characterized, triggers for the process *in vivo* are incompletely understood. It is not clear whether neutrophils release ETs as a specific response to stimuli *in vivo*, or if the presence of NETs is simply the aftermath of these cells being overwhelmed with inflammatory signals or pathogen insult, and/or if the mechanism for clearance of effete neutrophils is overwhelmed. Nonetheless, the fundamental outcome of NET formation in most studies is lysis of neutrophils and accompanying extracellular release of cytotoxic molecules. This outcome seemingly defies the numerous aforementioned host systems that are in place to ensure safe neutrophil removal and minimize damage to surrounding host tissues. Moreover, cell-free DNA and DNA-binding proteins (e.g., histones or high mobility group box 1 protein) – all components of NETs – are classic damage-associated molecular pattern autoantigens. NETs have been reported to activate and perpetuate the immune response and, thereby, promote chronic inflammation. Indeed, NET-associated molecules have been shown to elicit inflammatory responses mediated by toll-like receptors (TLRs), which may, in turn, impact autoimmunity (88). This topic has been reviewed recently by Thieblemont and colleagues (89).

Extracellular traps have been detected in a growing number of inflammatory and autoimmune diseases, in which contribution of neutrophils, or more specifically, cytotoxic components released during neutrophil lysis, was previously reported (Table 1). In these pathologic conditions, NETs appear harmful and sustain inflammatory processes. For example, Kolaczkowska et al. showed in an animal model of *S. aureus*-induced sepsis that extensive liver damage was primarily caused by neutrophil influx and presence of NETs within the liver vasculature (90). Necrotic liver damage was reduced significantly in mice deficient in neutrophil elastase or PAD4, as these mice had decreased ability to form NETs (90). NET components are also potent procoagulants that activate factor XII of the coagulation cascade and contribute to formation of both venous and arterial thrombi. Thus, NETs play an instrumental role in deep vein thromboses, atherosclerosis, or acute myocardial infarction (56, 91–97). In certain types of respiratory diseases, removal of NETs reduces some of the disease-associated symptoms. In lung diseases, in which NETs contribute to formation of obstructive “plugs,” human recombinant DNase I has been used to dismantle NETs (98–100). This treatment reduces the risk of disease exacerbation and improves overall outcome for the patient (98–100). Consistent with those findings, DNase treatment and removal of NETs has also been shown to improve lung function in murine asthma models (101).

**Table 1:** **Selected neutrophil-associated inflammatory diseases and contribution of NETs**.

Syndrome/disease	Description/role of neutrophils	Contribution of NETs	Reference
**Pulmonary disorders**			
Cystic fibrosis lung disease	Neutrophils contribute to many of the pathological manifestations of CF, including vigorous inflammation, chronic bacterial infections, and a self-perpetuating cycle of airway obstruction	CXCR2-mediated and NADPH oxidase-independent NET release	(102)
Chronic obstructive pulmonary disease (COPD)	Aberrant inflammatory response to cigarette smoke or other particles; emphysema	NETs and NETotic neutrophils are present in COPD sputum	(103, 104)
		NETs contribute to the severity of restricted airflow	
Respiratory syncytial virus disease (RSV)	Major cause of lower respiratory tract disease in children. Extensive neutrophil accumulation	Occlusion of small airways by DNA rich plugs. NETs have the ability to capture RSV particles	(105)
Acute lung injury (ALI) and acute respiratory distress syndrome (ARDS)	Involves complement C5 activation, acute inflammatory response and neutrophil accumulation, alveolar hemorrhage, edema, and fibrin deposition	NETs induce toxicity in epithelial and endothelial cells	(106, 107)
		Predominant role of histones in lung epithelial and endothelial cell death	
**Vascular disorders**			
Venous thromboembolism (VTE), including pulmonary embolism (PE) and deep vein thrombosis (DVT)	Inflammatory cells play a key role in thrombus formation; large numbers of neutrophils in early thrombus	NETs are present in the initial stage of thrombus formation	(56, 96, 97)
Disseminated intravascular coagulation (DIC)	Wide spread activation of coagulation; thrombotic occlusion of small and midsize vessels	NETs promote coagulation	(108)
Acute tubular necrosis, acute renal failure	Cell necrosis during initial inflammation, which amplifies the inflammatory response (renal necroinflammation)	NETs as a DAMP signal	(109)
Atherosclerosis	Chronic inflammation of the arterial wall. Neutrophil elastase-dependent secretion and activation of IL-1β by endothelial cells; LL-37	NETs present in atherosclerotic plaques and contribute to endothelium dysfunction	(43, 94, 110)
Acute myocardial infarction	Rupture of coronary atherosclerotic plaque and subsequent thrombotic occlusion of the vessel	NETs and histones as a pro-coagulant	(95)
Acute thrombotic microangiopathies (TMA)	Excessive microvascular thrombosis	Decreased DNase I activity leads to impaired NET degradation	(111)
Transfusion-related acute lung injury (TRALI)	Presence of anti-neutrophil antibodies. Activation of neutrophils in lungs that leads to damage of the endothelium and capillary leakage	Abundance of NETs in affected alveoli	(112)
Primary systemic vasculitis: granulomatosis with polyangiitis (GPA) (Wegener’s granulomatosis) and microscopic polyangiitis	Necrotizing vasculitis that affects small and medium size vessels – results in organ dysfunction; involvement of ANCA; neutrophilic inflammation; and formation of neutrophil granulomas	Not verified	(113, 114)
**Others**			
Systemic lupus erythematosus (SLE)	Systemic autoimmune disease characterized by production of autoantibodies against self-nuclear antigens; more apoptotic neutrophils in circulation	Patients develop antibodies against DNA and antimicrobial peptides present in NETs	(58, 115, 116)
		NETs increase the risk of venous and arterial thromboses	
		An abnormal subset of neutrophils, called low-density granulocytes (LDGs), are present in SLE. These cells form NETs readily, but a direct contribution to SLE remains to be determined	
Pancreatitis	Granulocytic epithelial lesions, formation of neutrophil rich aggregates and occlusion of pancreatic ducts	NET aggregates occlude pancreatic ducts and promote inflammation	(117)
Psoriasis	Immune-mediated genetic disorder; dysregulation between immune system and cutaneous cells, dendritic cells and lymphocytes are key players; characterized by hyperkeratotic plaques	Release of IL-17 during NET formation; subset of LDG similar to those in SLE; neutrophil elastase cleaves IL-36Ra, which is linked to psoriatic inflammation	(118–120)
Tumors (e.g., Ewing sarcoma, Lewis lung carcinoma; chronic myelogenous leukemia)	Not well defined; MMP-9 (gelatinase), cathepsin G, and neutrophil elastase contribute to tumor proliferation and angiogenesis	Primary tumors facilitate NET production from circulating neutrophils	(121–124)
		NETs can influence proliferation of B cells	
Liver metastases after surgical stress	Activation of immune system after surgery, which enhances the risk of systemic metastases and tumor recurrences	Production of NETs activates TLR9 pathway to induce their pro-tumorigenic activity	(125)
Periodontitis	Chronic inflammation of periodontium that is triggered by bacterial infection and subsequent influx of neutrophils	NETs present	(126)
Rheumatoid arthritis (RA)	Systemic autoimmune disease, which has genetic and environment risk factors; joint inflammation and damage mediated by influx of immune cells into synovial joint space. Cartilage destruction mediated by ROS production and secretion of proteases	Increased spontaneous NETosis	(60, 127)
		NETs as targets for auto-antibody	
Inflammatory bowel diseases (IBD) includes Crohn’s disease (CD) and ulcerative colitis (UC)	Chronic relapsing gastrointestinal inflammation	Possible induction of NETs through NOX2 (gp91*phox*)	(128, 129)
Chronic otitis media (COM)	Acute middle ear infection that can result in hearing loss; characterized by mucoid effusions	NETs play a central role in effusions	(130)
Gout (form of arthritis)	Precipitation of uric acid induces rapid onset of inflammation and influx of neutrophils into affected joint	Possibly anti-inflammatory mediators	(131)

## Concluding Perspective

Formation of NETs is usually accompanied by neutrophil lysis, although there are notable exceptions (86). Here, we focus our discussion solely on NETs that form following neutrophil lysis. A cytolytic process for NET formation exposes the host to toxic molecules that contribute to inflammation, tissue damage, and disease. Inasmuch as the potential for neutrophil lysis poses a significant threat to human health, neutrophil activation and turnover are highly regulated. Multiple host mechanisms exist to prevent neutrophil lysis and control release of cytotoxic granule components and ROS – and these regulatory processes are presumably circumvented by the formation of NETs. Therefore, it seems unlikely that the host immune system has evolved to use NETs as routine means for innate host defense against microbes. Rather, we suggest formation of NETs by cytolysis is an incidental phenomenon and not a *standard* or *traditional* means used by neutrophils to eliminate invading microorganisms. Such a hypothesis is more consistent with neutrophil biology and function, including recent studies of phagocytosis (132), and has no bearing on NET function *per se*. In other words, NETs may simply be the remnants of dead neutrophils – however effective they may be at ensnaring and/or killing microbes. On the other hand, a mechanism of NET formation that leaves neutrophils intact – as with vital NET formation – avoids many of the caveats of a cytolytic process and merits further investigation.

## Author Contributions

NM, SK, MQ, and FD wrote and edited the manuscript.

## Conflict of Interest Statement

The authors declare that the research was conducted in the absence of any commercial or financial relationships that could be construed as a potential conflict of interest.
